# Real-world incidence of endopthalmitis after intravitreal anti-VEGF injections in Korea: findings from the Common Data Model in ophthalmology

**DOI:** 10.4178/epih.e2021097

**Published:** 2021-11-09

**Authors:** Yongseok Mun, Seng Chan You, Da Yun Lee, Seok Kim, Yoo-Ri Chung, Kihwang Lee, Ji Hun Song, Young Gun Park, Young Hoon Park, Young-Jung Roh, Se Joon Woo, Kyu Hyung Park, Rae Woong Park, Sooyoung Yoo, Dong-Jin Chang, Sang Jun Park

**Affiliations:** 1 Department of Ophthalmology, Kangnam Sacred Heart Hospital, Hallym University College of Medicine, Seoul, Korea; 2 Department of Preventive Medicine, Yonsei University College of Medicine, Seoul, Korea; 3 Department of Ophthalmology, Seoul National University College of Medicine, Seoul National University Bundang Hospital, Seongnam, Korea; 4 Healthcare Information & Communications Technology (ICT) Research Center, Office of eHealth Research and Businesses, Seoul National University Bundang Hospital, Seongnam, Korea; 5 Department of Ophthalmology, Ajou University School of Medicine, Suwon, Korea; 6 Department of Ophthalmology and Visual Science, Seoul St. Mary’s Hospital, College of Medicine, The Catholic University of Korea, Seoul, Korea; 7 Eye Hospital, Yeouido St. Mary’s Hospital, College of Medicine, The Catholic University of Korea, Seoul, Korea; 8 Department of Biomedical Informatics, Ajou University School of Medicine, Suwon, Korea

**Keywords:** Anti-vascular endothelial growth factor, Endophthalmitis, Intravitreal injections, Common Data Model

## Abstract

**OBJECTIVES:**

The aim of this study was to evaluate the real-world incidence of endophthalmitis after intravitreal anti-vascular endothelial growth factor (VEGF) injections using data from the Observational Medical Outcomes Partnership (OMOP) Common Data Model (CDM).

**METHODS:**

Patients with endophthalmitis that developed within 6 weeks after intravitreal anti-VEGF injections were identified in 3 large OMOP CDM databases.

**RESULTS:**

We identified 23,490 patients who received 128,123 intravitreal anti-VEGF injections. The incidence rates of endophthalmitis were 15.75 per 10,000 patients and 2.97 per 10,000 injections. The incidence rates of endophthalmitis for bevacizumab, ranibizumab, and aflibercept (per 10,000 injections) were 3.64, 1.39, and 0.76, respectively. The annual incidence has remained below 5.00 per 10,000 injections since 2011 despite the increasing number of intravitreal anti-VEGF injections. Bevacizumab presented a higher incidence rate for endophthalmitis than ranibizumab and aflibercept (incidence rate ratio, 3.17; p=0.021).

**CONCLUSIONS:**

The incidence of endophthalmitis after intravitreal anti-VEGF injections has stabilized since 2011 despite the explosive increase in anti-VEGF injections. The off-label use of bevacizumab accounted for its disproportionately high incidence of endophthalmitis. The OMOP CDM, which includes off-label uses, laboratory data, and a scalable standardized database, could provide a novel strategy to reveal real-world evidence, especially in ophthalmology.

## INTRODUCTION

Anti-vascular endothelial growth factor (VEGF) drugs have revolutionized the treatment of causes of blindness, including age-related macular degeneration and diabetic retinopathy [1]. They also play an essential therapeutic role in cases of retinal vein occlusion, neovascular glaucoma, and retinopathy of prematurity [2,3]. In recent years, ranibizumab, aflibercept, and off-label bevacizumab have been widely used [4-6]. Several well-designed studies have shown the efficacy of anti-VEGF drugs for the aforementioned indications. Despite the efficacy and increased usage of these drugs, anti-VEGF agents have the potential risk of causing infectious endophthalmitis, sterile uveitis, retinal detachment, vitreous hemorrhage, and increased intraocular pressure [7]. Of these, infectious endophthalmitis is a devastating and sight-threatening complication that occurs after intravitreal anti-VEGF injections [8]. Therefore, studies analyzing electronic health record (EHR) data, claims data, and real-world registries or databases have been reported [9,10].

In the present study, we used the Observational Medical Outcomes Partnership (OMOP) Common Data Model (CDM) databases at several large institutions in Korea to estimate the incidence of infectious endophthalmitis following intravitreal anti-VEGF injections and studied the characteristics of patients who developed infectious endophthalmitis.

## MATERIALS AND METHODS

### Observational Medical Outcomes Partnership Common Data Model and vocabulary mapping

The deployment of the OMOP CDM in 2010 advanced the science of active medical product safety surveillance using observational healthcare data [11]. The OMOP CDM has been further developed through the Observational Health Data Science and Informatics (OHDSI) collaboration to conduct research across disparate observational healthcare databases in a distributed research network. The OMOP CDM adopted standardized structures, contents, and semantics of observational data. This feature permits researchers to share their analysis codes at every site when their healthcare data are properly extracted, transformed, and loaded (ETL) in accordance with the OMOP CDM. The OHDSI also provides a standardized way to define and generate phenotypes through an open-source software stack known as Atlas; it also has validated methodologies to aggregate results from multiple network sites into a single answer to produce real-world evidence [12]. In terms of standardization, the OMOP CDM introduces common conventions called “standard vocabularies” into its system for integrating patient-level data from heterogeneous data sources across multiple sites [13]. This is a kind of mapping based on the individual code system used for diagnosis, drug usage, laboratory results, and other clinical measurements using standard nomenclature systems as follows: Systematized Nomenclature of Medicine, Clinical Terms (SNOMED CT) for diagnosis, RxNorm and the RxNorm extension for drugs, and Logical Observation Identifiers Names and Codes (LOINC) for laboratory results and other clinical measurements [14-16]. This mapping process, developed by the working group of the OHDSI community, is shared to standardize mapping [17]. We mapped the drugs used in our study to the RxNorm vocabulary (Supplementary Material 1, which demonstrates concept identifiers for 3 anti-VEGF drugs and 2 antibiotics). We also verified drug codes manually to identify that they encoded the same drugs across the 3 hospitals. Despite the ongoing efforts by the OHDSI working group, most ophthalmologic measurements, such as optical coherence tomography (OCT) or ophthalmic biometry, are currently not available under the OMOP CDM. Recently, several working groups have attempted to create standard ophthalmologic vocabularies and ETL processes for these data.

### Database and study population

We analyzed data from 3 OMOP CDM databases of 3 large tertiary referral hospitals—Seoul National University Bundang Hospital (SNUBH), Seoul St. Mary’s Hospital (SSHM), and Ajou University Hospital (AUH)—which are among the largest hospitals in Korea. These 3 EHR-based OMOP CDM databases included 1,734,565, 3,219,111, and 3,109,677 patients, respectively (accessed on July 1, 2020) (Table 1). These OMOP CDM databases were encoded in the OMOP CDM version 5.3.1.

**Figure 1.**
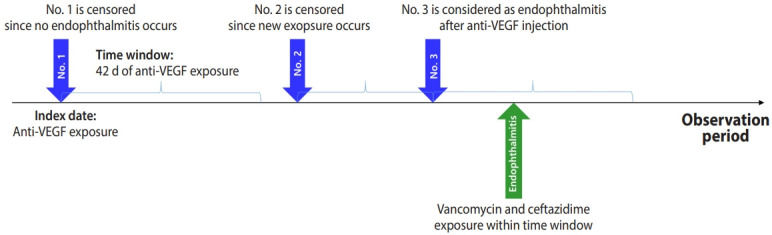
The definition of endophthalmitis occurrence among patients receiving intravitreal anti-vascular endothelial growth factor (VEGF) injections.

We did not use route concept identification to distinguish intravitreal injections. Instead, we considered anti-VEGF drugs (bevacizumab, ranibizumab, and aflibercept) prescribed by ophthalmologists as intravitreal injections since intravenous anti-VEGF drugs are used for chemotherapy (https://github.com/ohdsi-korea/ThemisKorea/wiki/PROVIDER). We generated retrospective cohorts, which included all events of exposure to any anti-VEGF drugs prescribed by ophthalmologists in the study period from January 1, 2007, to December 31, 2018, in each of the 3 databases. We assessed all intravitreal anti-VEGF injections among the patients included in this study. The index date was defined as the date of each intravitreal anti-VEGF injection (Figure 1). All ophthalmologists followed the guidelines for intravitreal injections [18].

### Identification of endophthalmitis occurrence and statistical analysis

In this retrospective cohort, we investigated the incidence of infectious endophthalmitis following intravitreal anti-VEGF injections. Infectious endophthalmitis requires empirical antibiotics since it is an urgent condition causing very severe visual deterioration. A combination of intravitreal vancomycin (1 mg/0.1 mL) and ceftazidime (2.25 mg/0.1 mL) is widely accepted as standard care for infectious endophthalmitis since these antibiotics cover a broad range of pathogens [19,20]. Ophthalmologists rarely prescribe these antibiotics intravenously together. Instead, they prescribe them intravitreally at the same time only to treat infectious endophthalmitis. Endophthalmitis was considered to occur at the time of the intravitreal injection; however, we allowed an interval of 6 weeks between the intravitreal injection and endophthalmitis treatment due to delayed symptoms. Therefore, endophthalmitis could be defined as any event wherein ophthalmologists prescribed both vancomycin and ceftazidime within 6 weeks of an intravitreal anti-VEGF injection [7,10]. The primary outcome was the incidence of infectious endophthalmitis over a 12-year period. The incidence rates of endophthalmitis were stratified for each drug, indication, and year. We also evaluated the incidence rate ratio (IRR) and its 95% confidence interval (CI) of each antiVEGF drug by comparing it with the others using Poisson regression. The name of each referral center was anonymized for research and confidentiality purposes. We shared only summary measures without any patient-level data. The statistical analysis was performed using R version 3.6.3 (R Foundation for Statistical Computing, Vienna, Austria).

### Ethics statement

This study was conducted following the tenets of the Declaration of Helsinki. Each center obtained approval from the institutional review board, and the waiver of informed consent was also approved since the OMOP CDM contains only de-identified data (IRB No. SNUBH, SSMH and AUH: X-1907-555-904, 2020-0806-0001 and RB-MED-MDB-20-271, respectively).

## RESULTS

We analyzed data from 8,063,353 patients across the 3 OMOP CDM databases, from which we identified a total of 128,123 intravitreal anti-VEGF injections in 23,490 patients (12,726 males, 54.2%) (Table 2). The mean number of injections per patient was 5.45. The mean age at the time of receipt of the first anti-VEGF injection was 62.4± 13.7 years. The most frequently injected drug was bevacizumab (n= 93,281, 72.8%), followed by ranibizumab (n= 21,598, 16.9%) and aflibercept (n= 13,244, 10.3%).

**Figure 2.**
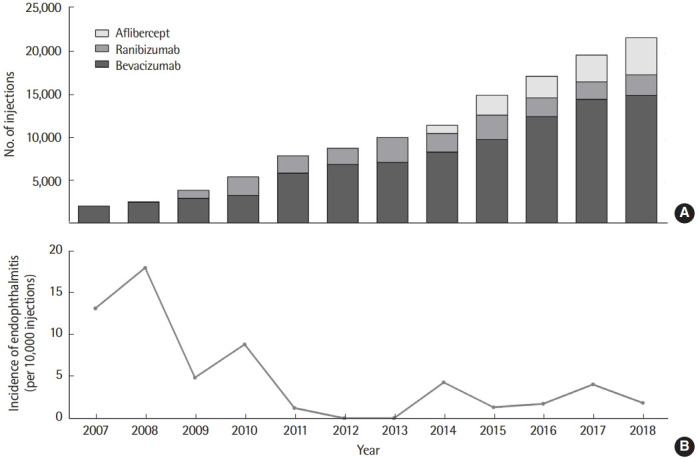
The number of anti-vascular endothelial growth factor injections (A) and incidence of endophthalmitis (B) in each year from 2007 to 2018.

We identified 38 cases of endophthalmitis occurring in 37 patients (27 males, 73.0%), and the incidence rate was 15.75 per 10,000 patients and 2.97 per 10,000 injections (Table 2). Two independent events occurred in 1 eye with a sufficient time interval. Of the 38 endophthalmitis cases occurring after intravitreal injections, 34 cases (89.5%) occurred after bevacizumab, 3 cases (7.9%) after ranibizumab, and 1 case (2.6%) after aflibercept, respectively. The incidence rate per 10,000 injections was 3.64 for bevacizumab, 0.14 for ranibizumab, and 0.76 for aflibercept, respectively (Table 3). Bevacizumab was significantly associated with endophthalmitis compared to non-bevacizumab drugs, including ranibizumab and aflibercept (IRR, 3.17; 95% CI, 1.13 to 8.95; p= 0.021). Bevacizumab showed an IRR of 2.62 (95% CI, 0.81 to 8.54; p= 0.096) compared with ranibizumab and an IRR of 4.83 (95% CI, 0.66 to 35.3; p= 0.086) compared with aflibercept. Ranibizumab showed an IRR of 1.84 (95% CI, 0.19 to 17.70; p= 0.590) compared with aflibercept. Though the number of intravitreal anti-VEGF injections has been increasing over the years, the incidence rate of endophthalmitis has tended to decrease (Figure 2). The annual incidence rate has remained below 5.00 per 10,000 injections since 2011 (Table 3).

At Center A, 36,460 intravitreal anti-VEGF injections were administered to 6,495 patients (3,552 males, 54.5%) were identified. The average number of injections per patient was 5.61. The mean age at the time of the first anti-VEGF injection was 63.5±14.0 years. Eight cases of endophthalmitis were observed in 8 eyes, and the incidence rate was 12.32 per 10,000 patients and 2.19 per 10,000 injections. At Center B, 66,185 intravitreal anti-VEGF injections were administered to 12,319 patients (6,508 males, 52.8%). The average number of injections per patient was 5.37. The mean age at the first anti-VEGF injection was 63.1 ± 13.3 years. Twenty-six cases of endophthalmitis were observed in 26 eyes, and the incidence rate was 21.1 per 10,000 patients and 3.93 per 10,000 injections. At Center C, 25,478 intravitreal anti-VEGF injections were administered to 4,676 patients (2,676 males, 57.2%). The average number of injections per patient was 5.45. The mean age at the first anti-VEGF injection was 59.0± 14.1 years. Four cases of endophthalmitis were observed in 3 eyes, and the incidence rate was 6.42 per 10,000 patients and 1.57 per 10,000 injections. The detailed characteristics of each population cohort are provided in Supplementary Materials 2 and 3, which demonstrate the demographics of the study population and patients with endophthalmitis at each participating center, respectively). No cases of endophthalmitis occurred after ranibizumab or aflibercept injections at Centers A and C (Supplementary Materials 3 and 4), which demonstrate the demographic characteristics of patients with endophthalmitis at each participating center and the incidence of infectious endophthalmitis stratified by year and drugs for each participating center, respectively).

## DISCUSSION

This study identified 38 cases of endophthalmitis in 37 patients following anti-VEGF injections (128,123 injections), among the 23,490 patients reviewed from 3 large-scale data sources. The incidence rate of endophthalmitis following anti-VEGF injections was 15.75 per 10,000 patients and 2.97 per 10,000 injections, which is similar to that reported by previous studies with large numbers of participants [9,21]. The annual incidence has remained below 5.00 per 10,000 injections since 2011, although the number of intravitreal anti-VEGF injections has been increasing. The incidence of endophthalmitis was the highest in patients who received bevacizumab injections (3.64 per 10,000 injections). Bevacizumab showed a significant association with endophthalmitis relative to other drugs (combining ranibizumab and aflibercept), which was also reported by Xu et al. [20]. Interestingly, Kiss et al. [9] showed a higher incidence of endophthalmitis with aflibercept than with bevacizumab or ranibizumab. VanderBeek et al. [22] demonstrated no significant difference between bevacizumab and ranibizumab. Since a vial of bevacizumab is divided into individual aliquots prior to administration, this division process might pose a risk of contamination, subsequently leading to endophthalmitis [23]. This explanation could possibly support the high incidence of endophthalmitis after bevacizumab injection in our study. In addition, we presumed that outbreaks occurred at Centers A and B due to their higher incidence rate of endophthalmitis following intravitreal bevacizumab (Supplementary Material 4), which demonstrates the incidence of infectious endophthalmitis stratified by year and drugs for each participating center). Except for bevacizumab, the incidence rates at all centers were very low and not significantly different from each other. The incidence of post-injection endophthalmitis has recently decreased substantially due to efforts to maintain a more aseptic environment, such as the introduction of prefilled syringes.

Kiss et al. [9] reported that vancomycin and ceftazidime were the most frequently administered antibiotics. Dossarps et al. [21] and Xu et al. [20] also conducted studies using intravitreal vancomycin and ceftazidime to manage infectious endophthalmitis. The use of 2 antibiotics to define endophthalmitis is quite reasonable since using diagnosis codes to identify patients with endophthalmitis involves factors such as coding accuracy; this strategy, however, guarantees robustness of the study. Factors such as contamination, anti-VEGF drug lots, or batches contribute to endophthalmitis following anti-VEGF injection [9]. Applying povidone-iodine to the ocular surface before an injection is the most effective method to prevent infection [24]. The use of a speculum or prefilled syringes also decreases the risk of infection; however, topical antibiotics do not contribute to risk reduction [24]. As shown in Figure 2, the incidence of endophthalmitis has tended to decrease over time, suggesting that clinicians have made efforts to reduce the risk factors for endophthalmitis.

The importance of real-world evidence derived from realworld data (RWD) has recently emerged, since conventional clinical trials have limitations that make it challenging to extend their findings to generalized populations beyond their highly controlled target participants [25]. Therefore, with increasing accessibility to digital health data, such as EHRs, billing data, and administrative data, the process of collecting and analyzing RWD has become very important [26]. The OMOP CDM, currently maintained by the OHDSI, enables the integration of RWD from multiple and heterogeneous forms of clinical data using common conventions and structures to represent the data [27]. In addition, this data model enables the analysis of RWD from different hospitals or institutions in a consistent way [12]. As of August 2019, the OHDSI has established a data network of over 100 different healthcare databases from over 20 countries, collectively capturing over 1 billion patient records by applying the OMOP CDM. It has included over 2,500 collaborators on its online forums from various stakeholders, such as academia, the medical product industry, regulators, government, payers, technology providers, health systems, clinicians, patients, and other representatives of different disciplines [12]. Several influential studies using the OMOP CDM have recently been published in fields other than ophthalmology [28-30].

In the present study, we introduced the OMOP CDM as a distributed research platform and showed how the OMOP CDM could effectively generate real-world evidence from RWD. Various methodologies are used by researchers to evaluate real-world evidence based on large populations, such as clinical registries, post-marketing surveillance, and claims data. These are reliable data sources, but there are a few points to consider. Clinical registries and post-marketing surveillance need additional efforts to build and manage their own cohorts, and they are less applicable to studies other than those originally intended. In claims data, items not covered by insurance may not be included. For example, the national health insurance service database lacks information on off-label bevacizumab use because it does not cover its use. Incorrect coding, coding variation, and coding overlap are also unavoidable problems of claims data [31]. Furthermore, claims data do not include laboratory results. Some information may be missed because of specific aspects of the billing process [31]. The OMOP CDM can help researchers by making it possible to integrate data sources originating from domestic and multiple institutions or hospitals worldwide as well, since it shares common structures. It also contains an ample amount of clinical information such as laboratory results, various measures, and information about diagnosis or drug usage across all departments, not limited to a specific entity. Our study demonstrated the realworld incidence of endophthalmitis following intravitreal antiVEGF injections and revealed the importance and usefulness of the OMOP CDM to develop real-world evidence. In the ophthalmologic field, this methodology has been lacking, and the present study has opened up possibilities for scalable research.

This study has several limitations. First, we cannot guarantee that all endophthalmitis patients visited the hospitals where they received intravitreal anti-VEGF injections. However, it would be very unlikely for endophthalmitis patients to visit other hospitals due to an absence of records of their treatment history. Second, our study was not population-based. The national health claims database of Korea, usually used in population-based studies, was not suitable since the subjects of our study were not the general population, but patients who received intravitreal anti-VEGF injections. Furthermore, the national health claims database does not cover the off-label use of intravitreal bevacizumab. Therefore, we conducted a multicenter observational study including 3 of the largest tertiary referral hospitals using the OMOP CDM, which could cover off-label prescriptions including intravitreal bevacizumab. Moreover, previous studies reported that the occurrence of endophthalmitis following intravitreal anti-VEGF injections did not depend on age, sex, or systemic comorbidities. Instead, it depended on the type of anti-VEGF drug or intravitreal injection procedures, such as povidone-iodine installation, squeezing or moving by the patient during the injection, or the type of anesthesia [7,9,18]. Since all ophthalmologists in our study followed intravitreal injection guidelines, the characteristics related to intravitreal injections in our patients could reflect that of the entire population. Therefore, we could not precisely calculate the population-based incidence rate using the OMOP CDM. In addition, we could not assume proportional hazards, since post-injection endophthalmitis was considered to occur at the time of the intravitreal injection. An interval of 6 weeks after the intravitreal injection was used as the time lag for delayed symptoms. Thus, we did not adjust the incidence rates by age, sex, or comorbidities or conduct a Cox proportional hazard regression analysis. Although the incidence rate was higher in males than females in this study, this finding should be interpreted with caution. Further studies including many regional-based hospitals are needed since only 3 tertiary referral hospitals in the capital region were included in our study. Third, other ophthalmologic data, such as visual acuity and intraocular pressure were not included in this analysis. As the investigations required to distinguish between sterile and infectious endophthalmitis, such as vitreous cultures, were not performed in this study, some patients might have had sterile endophthalmitis rather than infectious endophthalmitis. In addition, only 3 centers participated in our study. These limitations all occurred because of a lack of consensus about encoding ophthalmologic data. Recently, several working groups have discussed the above aspects in earnest. Furthermore, the ETL processes of various ophthalmic examinations such as OCT, ophthalmic biometry, and the Humphrey visual field are ongoing. We have already conducted additional real-world studies about various ophthalmic diseases and are ready to report them. We look forward to other real-world studies using OMOP CDM with those images and numeric data from ophthalmic examinations. In that case, more institutions will participate in distributed research using OMOP CDM in the ophthalmologic field.

In conclusion, this retrospective, multicenter cohort study shows that the incidence of endophthalmitis in patients following anti-VEGF injection was very low and comparable with those reported by previous studies of large populations. To the best of our knowledge, this is the first study to reveal real-world evidence using OMOP CDM in ophthalmology. This methodology may facilitate large-scale collaborative research for real-world evidence and is expected to be widely used in ophthalmology.

## Figures and Tables

**Table 1.** Characteristics of the population included in the OMOP CDM databases from 3 tertiary referral hospitals

Data source	No. of patients	Male	Female
Center A	1,734,565	828,617 (47.8)	905,948 (52.2)
Center B	3,219,111	1,541,620 (47.9)	1,677,491 (52.1)
Center C	3,109,677	1,649,109 (53.0)	1,460,568 (47.0)
Total no. of patients	8,063,353	4,019,346 (49.8)	4,044,007 (50.1)

Values are presented as number (%). OMOP, Observational Medical Outcomes Partnership; CDM, Common Data Model.

**Table 2.** Demographic characteristics of patients treated with intravitreal anti-vascular endothelial growth factor (VEGF) and endophthalmitis cases from 2007 to 2018

Characteristics		Total				Endophthalmitis			
		All drugs	Bevacizumab	Ranibizumab	Aflibercept	All drugs	Bevacizumab	Ranibizumab	Aflibercept
No. of patients		23,490	20,901	4,498	2,804	37	33	3	1
No. of injections		128,123	93,281	21,598	13,244	38	34	3	1
Sex, male		12,726 (54.2)	11,258 (53.9)	2,526 (56.2)	1,741 (62.1)	27 (73.0)	25 (75.8)	2 (66.7)	0 (0.0)
Age at first anti-VEGF injection		62.4±13.7	61.6±13.7	69.3±10.8	68.9±10.9	61.6±16.1	61.7±16.0	74.7±13.0	74.2±0.0
Comorbidities									
	Diabetes mellitus	5,649 (24.0)	5,356 (25.6)	709 (15.8)	443 (15.8)	17 (45.9)	17 (51.5)	0 (0.0)	0 (0.0)
	Hypertension	3,688 (15.7)	3,335 (16.0)	677 (15.1)	464 (16.5)	1 (2.7)	1 (3.0)	0 (0.0)	0 (0.0)
	Ischemic heart disease	1,305 (5.6)	1,152 (5.5)	266 (5.9)	186 (6.6)	1 (2.7)	1 (3.0)	0 (0.0)	0 (0.0)
	Atrial fibrillation	261 (1.1)	228 (1.1)	67 (1.5)	42 (1.5)	1 (2.7)	1 (3.0)	0 (0.0)	0 (0.0)
	Cerebrovascular disease	1,713 (7.3)	1,559 (7.5)	416 (9.2)	253 (9.0)	3 (8.1)	3 (9.1)	0 (0.0)	0 (0.0)
	Cancer	1,797 (7.7)	1,569 (7.5)	401 (8.9)	295 (10.5)	0 (0.0)	0 (0.0)	0 (0.0)	0 (0.0)
	Chronic kidney disease	1,247 (5.3)	1,198 (5.7)	102 (2.3)	85 (3.0)	4 (10.8)	4 (12.1)	0 (0.0)	0 (0.0)
	Chronic obstructive pulmonary disease	253 (1.1)	213 (1.0)	76 (1.7)	52 (1.9)	0 (0.0)	0 (0.0)	0 (0.0)	0 (0.0)

Values are presented as mean±standard deviation or number (%).

**Table 3.** Incidence of endophthalmitis after anti-vascular endothelial growth factor injections by drugs, indications, and year

Variables		No. of injections		
		Without endophthalmitis	With endophthalmitis	Incidence (per 10,000 injections)
Drug				
	Bevacizumab (2.00 mg/0.05 mL)	93,247	34	3.64
	Ranibizumab (1.25 mg/0.05 mL)	21,595	3	1.39
	Aflibercept (0.50 mg/0.05 mL)	13,243	1	0.76
Indication				
	Neovascular age-related macular degeneration	58,790	11	1.87
	Retinal vein occlusion	7,013	3	4.28
	Diabetic macular edema	11,843	3	2.53
	Others	50,439	21	4.16
Year				
	2007	2,287	3	13.11
	2008	2,782	5	17.97
	2009	4,126	2	4.84
	2010	5,674	5	8.80
	2011	8,116	1	1.23
	2012	8,992	0	0.00
	2013	10,258	0	0.00
	2014	11,674	5	4.28
	2015	15,170	2	1.32
	2016	17,350	3	1.73
	2017	19,807	8	4.04
	2018	21,849	4	1.83
	Overall	128,085	38	2.97

## SUPPLEMENTARY MATERIALS

Supplementary Material 1. Concept identifiers for 3 anti-vascular endothelial growth factor drugs and 2 antibiotics

Supplementary Material 2. Demographic characteristics of the study population at each participating center

Supplementary Material 3. Demographic characteristics of patients with endophthalmitis at each participating center

Supplementary Material 4. Incidence of infectious endophthalmitis after intravitreal anti-vascular endothelial growth factor injections stratified by year and drugs for each participating center
