# Supplementary material for: Real-world incidence of endopthalmitis after intravitreal anti-VEGF injections in Korea: findings from the Common Data Model in ophthalmology
Source: Epidemiol Health. 2021 Nov 9;43:e2021097. doi: 10.4178/epih.e2021097 (PMC8864106; doi:10.4178/epih.e2021097)
Supplement: Supplementary Material 1. — Concept identifiers for 3 anti-vascular endothelial growth factor drugs and 2 antibiotics [file epih-43-e2021097-suppl1.docx]

**Supplementary Material 1. Concept identifiers for 3 anti-vascular endothelial growth factor drugs and 2 antibiotics**

| **Drug** | **Concept Identifier** | **Concept Name** | **Standard Vocabulary** |
| --- | --- | --- | --- |
| Bevacizumab | 1397141 | Bevacizumab | RxNorm |
| Ranibizumab | 19080982 | Ranibizumab | RxNorm |
| Aflibercept | 40244266 | Aflibercept | RxNorm |
| Vancomycin | 1707687 | Vancomycin | RxNorm |
| Ceftazidime | 1776684 | Ceftazidime | RxNorm |
