# Supplementary material for: Real-world incidence of endopthalmitis after intravitreal anti-VEGF injections in Korea: findings from the Common Data Model in ophthalmology
Source: Epidemiol Health. 2021 Nov 9;43:e2021097. doi: 10.4178/epih.e2021097 (PMC8864106; doi:10.4178/epih.e2021097)
Supplement: Supplementary Material 2. — Demographic characteristics of the study population at each participating center [file epih-43-e2021097-suppl2.docx]

**Supplementary Material 2. Demographic characteristics of the study population at each participating center**

|  | | **Center A** | | | | **Center B** | | | | **Center C** | | | |
| --- | --- | --- | --- | --- | --- | --- | --- | --- | --- | --- | --- | --- | --- |
|  | | All drugs | Bevacizumab | Ranibizumab | Afilbercept | All drugs | Bevacizumab | Ranibizumab | Afilbercept | All drugs | Bevacizumab | Ranibizumab | Afilbercept |
| Number of patients | | 6,495 | 5,557 | 1424 | 964 | 12,319 | 11,019 | 2,451 | 1,527 | 4,676 | 4325 | 623 | 313 |
| Male (%) | | 3,542 (54.5%) | 3,018 (54.3%) | 799 (56.1%) | 590 (61.2%) | 6,508 (52.8%) | 5,780 (52.5%) | 1,361 (55.5%) | 951 (62.3%) | 2,676 (57.2%) | 2,460 (56.9%) | 366 (58.7%) | 200 (63.9%) |
| Age at first anti-VEGF injection (Mean±SD) | | 63.5 ± 14.0 | 62.7 ± 14.1 | 69.4 ± 11.5 | 70.1 ± 11.1 | 63.1 ± 13.3 | 62.5 ± 13.4 | 69.5 ± 10.4 | 68.9 ± 10.6 | 59.0 ± 14.1 | 58.0 ± 14.1 | 68.0 ± 10.6 | 65.0 ± 11.5 |
|  | Male | 62.7 ± 13.8 | 61.8 ± 13.8 | 69.2 ± 11.1 | 69.9 ± 10.9 | 63.7 ± 13.6 | 61.9 ± 13.2 | 68.9 ± 9.9 | 68.7 ± 10.3 | 57.0 ± 13.9 | 56.0 ± 13.7 | 68.0 ± 10.5 | 66.0 ± 10.7 |
|  | Female | 64.4 ± 14.3 | 63.8 ± 14.4 | 69.8 ± 12.0 | 70.4 ± 11.3 | 62.5 ± 13.0 | 63.3 ± 13.6 | 70.3 ± 11.0 | 69.2 ± 11.1 | 61.0 ± 14.3 | 60.0 ± 14.4 | 69.0 ± 10.6 | 65.0 ± 12.9 |
| Comorbidities | |  |  |  |  |  |  |  |  |  |  |  |  |
|  | Diabetes mellitus | 2,242 (34.5%) | 2,093 (37.7%) | 303 (21.3%) | 225 (23.3%) | 2,067 (16.78%) | 1,966 (17.8%) | 303 (12.4%) | 138 (9.0%) | 1,340 (28.7%) | 1,297 (30.0%) | 103 (16.5%) | 80 (25.6%) |
|  | Hypertension | 1,167 (18%) | 1,029 (18.5%) | 270 (19%) | 207 (21.5%) | 1,239 (10.06%) | 1,098 (10.0%) | 262 (10.7%) | 161 (10.5%) | 1,282 (27.4%) | 1,208 (27.9%) | 145 (23.3%) | 96 (30.7%) |
|  | Ischemic heart disease | 271 (4.2%) | 232 (4.2%) | 66 (4.6%) | 57 (5.9%) | 579 (4.70%) | 512 (4.7%) | 129 (5.3%) | 84 (5.50%) | 455 (9.7%) | 408 (9.4%) | 71 (11.4%) | 45 (14.4%) |
|  | Atrial fibrillation | 116 (1.8%) | 99 (1.8%) | 39 (2.7%) | 17 (1.8%) | 105 (0.85%) | 89 (0.8%) | 26 (1.1%) | 25 (1.6%) | 40 (0.9%) | 40 (0.9%) | 2 (0.3%) | 0 (0.0%) |
|  | Cerebrovascular disease | 778 (12%) | 662 (11.9%) | 205 (14.4%) | 134 (13.9%) | 774 (6.29%) | 746 (6.8%) | 195 (8.0%) | 104 (6.8%) | 161 (3.4%) | 151 (3.5%) | 16 (2.6%) | 15 (4.8%) |
|  | Cancer | 747 (11.5%) | 617 (11.1%) | 204 (14.3%) | 184 (19.1%) | 460 (3.73%) | 410 (3.7%)_ | 120 (4.9%) | 74 (4.8%) | 590 (12.6%) | 542 (12.5%) | 77 (12.4%) | 37 (11.8%) |
|  | Chronic kidney disease | 367 (5.7%) | 352 (6.3%) | 43 (3%) | 35 (3.6%) | 385 (3.13%) | 363 (3.3%) | 36 (1.5%) | 28 (1.8%) | 495 (10.6%) | 483 (11.2%) | 23 (3.7%) | 22 (7%) |
|  | Chronic obstructive pulmonary disease | 70 (1.1%) | 66 (1.2%) | 23 (1.6%) | 18 (1.9%) | 97 (0.79%) | 73 (0.7%) | 29 (1.2%) | 26 (1.7%) | 86 (1.8%) | 74 (1.7%) | 24 (3.9%) | 8 (2.6%) |

VEGF = Vascular Endothelial Growth Factor
