# Supplementary material for: Real-world incidence of endopthalmitis after intravitreal anti-VEGF injections in Korea: findings from the Common Data Model in ophthalmology
Source: Epidemiol Health. 2021 Nov 9;43:e2021097. doi: 10.4178/epih.e2021097 (PMC8864106; doi:10.4178/epih.e2021097)
Supplement: Supplementary Material 3. — Demographic characteristics of patients with endophthalmitis at each participating center [file epih-43-e2021097-suppl3.docx]

**Supplementary Material 3. Demographic characteristics of patients with endophthalmitis at each participating center**

|  | | **All drugs** | | | **Bevacizumab** | | | **Ranibizumab** | | | **Aflibercept** | | |
| --- | --- | --- | --- | --- | --- | --- | --- | --- | --- | --- | --- | --- | --- |
| **Center** | | **A** | **B** | **C** | **A** | **B** | **C** | **A** | **B** | **C** | **A** | **B** | **C** |
| Age at first anti-VEGF injection (Mean±SD) | | 63.5 ± 12.5 | 60.7 ± 17.7 | 64.0 ± 12.4 | 63.5 ± 12.5 | 58.1 ± 17.7 | 64.0 ± 12.4 | - | 74.7 ± 13.0 | - | - | 74.2 | - |
| Male (%) | | 7 (87.5%) | 19 (73.1%) | 1 (33.3%) | 7 (87.5%) | 17 (77.3%) | 1 (33.3%) | - | 2 (66.7%) | - | - | 0 (0.0%) | - |
| Number of injections | | 8 | 26 | 3 | 8 | 22 | 3 | 0 | 3 | 0 | 0 | 1 | 0 |
| Number of eyes with endophthalmitis | | 8 | 26 | 4 | 8 | 22 | 4 | 0 | 3 | 0 | 0 | 1 | 0 |
| Comorbidities | |  |  |  |  |  |  |  |  |  |  |  |  |
|  | Diabetes mellitus | 2 | 15 | 0 | 2 | 15 | 0 | 0 | 0 | 0 | 0 | 0 | 0 |
|  | Hypertension | 1 | 0 | 0 | 1 | 0 | 0 | 0 | 0 | 0 | 0 | 0 | 0 |
|  | Ischemic heart disease | 0 | 1 | 0 | 0 | 1 | 0 | 0 | 0 | 0 | 0 | 0 | 0 |
|  | Atrial fibrillation | 0 | 1 | 0 | 0 | 1 | 0 | 0 | 0 | 0 | 0 | 0 | 0 |
|  | Cerebrovascular disease | 1 | 2 | 0 | 1 | 2 | 0 | 0 | 0 | 0 | 0 | 0 | 0 |
|  | Cancer | 0 | 0 | 0 | 0 | 0 | 0 | 0 | 0 | 0 | 0 | 0 | 0 |
|  | Chronic kidney disease | 0 | 4 | 0 | 0 | 4 | 0 | 0 | 0 | 0 | 0 | 0 | 0 |
|  | COPD | 0 | 0 | 0 | 0 | 0 | 0 | 0 | 0 | 0 | 0 | 0 | 0 |

VEGF = Vascular Endothelial Growth Factor; TIA = Transient Ischemic Attack; COPD = Chronic Obstructive Pulmonary Disease
