# Supplementary material for: Real-world incidence of endopthalmitis after intravitreal anti-VEGF injections in Korea: findings from the Common Data Model in ophthalmology
Source: Epidemiol Health. 2021 Nov 9;43:e2021097. doi: 10.4178/epih.e2021097 (PMC8864106; doi:10.4178/epih.e2021097)
Supplement: Supplementary Material 4. — Incidence of infectious endophthalmitis after intravitreal anti-vascular endothelial growth factor injections stratified by year and drugs for each participating center [file epih-43-e2021097-suppl4.docx]

**Supplementary Material 4. Incidence of infectious endophthalmitis after intravitreal anti-vascular endothelial growth factor injections stratified by year and drugs for each participating center**

|  | **Bevacizumab** | | | | | | **Ranibizumab** | | | | | | **Aflibercept** | | | | | |
| --- | --- | --- | --- | --- | --- | --- | --- | --- | --- | --- | --- | --- | --- | --- | --- | --- | --- | --- |
|  | **Without Endopthalmitis** | | | **With Endopthalmitis** | | | **Without Endopthalmitis** | | | **With Endopthalmitis** | | | **Without Endopthalmitis** | | | **With Endopthalmitis** | | |
| **Center** | **A** | **B** | **C** | **A** | **B** | **C** | **A** | **B** | **C** | **A** | **B** | **C** | **A** | **B** | **C** | **A** | **B** | **C** |
| 2007 | 673 | 1,614 | 0 | 2 | 1 | 0 | 0 | 0 | 0 | 0 | 0 | 0 | 0 | 0 | 0 | 0 | 0 | 0 |
| 2008 | 791 | 1,933 | 0 | 3 | 2 | 0 | 56 | 0 | 2 | 0 | 0 | 0 | 0 | 0 | 0 | 0 | 0 | 0 |
| 2009 | 991 | 2,159 | 0 | 0 | 2 | 0 | 352 | 522 | 102 | 0 | 0 | 0 | 0 | 0 | 0 | 0 | 0 | 0 |
| 2010 | 972 | 1,995 | 536 | 1 | 3 | 0 | 694 | 1,216 | 261 | 0 | 1 | 0 | 0 | 0 | 0 | 0 | 0 | 0 |
| 2011 | 1,129 | 2,948 | 2,019 | 1 | 0 | 0 | 613 | 1,045 | 362 | 0 | 0 | 0 | 0 | 0 | 0 | 0 | 0 | 0 |
| 2012 | 1,249 | 3,523 | 2,344 | 0 | 0 | 0 | 647 | 1,005 | 224 | 0 | 0 | 0 | 0 | 0 | 0 | 0 | 0 | 0 |
| 2013 | 1,255 | 3,871 | 2,229 | 0 | 0 | 0 | 955 | 1,558 | 369 | 0 | 0 | 0 | 0 | 21 | 0 | 0 | 0 | 0 |
| 2014 | 1,373 | 4,786 | 2,409 | 0 | 3 | 1 | 732 | 1,182 | 229 | 0 | 1 | 0 | 278 | 551 | 134 | 0 | 0 | 0 |
| 2015 | 1,799 | 5,339 | 2,852 | 0 | 1 | 0 | 1,021 | 1,417 | 418 | 0 | 1 | 0 | 781 | 1,315 | 228 | 0 | 0 | 0 |
| 2016 | 3,060 | 6,553 | 3,045 | 0 | 2 | 1 | 830 | 920 | 440 | 0 | 0 | 0 | 1,054 | 1,226 | 222 | 0 | 0 | 0 |
| 2017 | 5,035 | 7,253 | 2,386 | 1 | 5 | 1 | 639 | 972 | 418 | 0 | 0 | 0 | 1,283 | 1,608 | 213 | 0 | 1 | 0 |
| 2018 | 5,893 | 6,103 | 3,130 | 0 | 3 | 1 | 634 | 1,343 | 417 | 0 | 0 | 0 | 1,663 | 2,181 | 485 | 0 | 0 | 0 |
